# Developing and Validating Tests of Reading and Listening Comprehension for Fifth and Sixth Grade Students in Portugal

**DOI:** 10.3389/fpsyg.2020.610876

**Published:** 2020-12-09

**Authors:** Bruna Rodrigues, Irene Cadime, Fernanda Leopoldina Viana, Iolanda Ribeiro

**Affiliations:** ^1^Psychology Research Centre, School of Psychology, University of Minho, Braga, Portugal; ^2^Research Centre on Child Studies, Institute of Education, University of Minho, Braga, Portugal; ^3^School of Psychology, University of Minho, Braga, Portugal

**Keywords:** reading comprehension, listening comprehension, Rasch model, vertical equating, validity evidence

## Abstract

An efficient assessment of reading and linguistic abilities in school children requires reliable and valid measures. Moreover, measures which include specific test forms for different academic grade levels, that are vertically equated, allow the direct comparison of results across multiple time points and avoid floor and ceiling effects. Two studies were conducted to achieve these goals. The purpose of the first study was to develop tests of reading and listening comprehension in European Portuguese, with vertically scaled test forms for students in the fifth and sixth grades, using Rasch model analyses. The purpose of the second study was to collect evidence for the validity of these tests based on the relationships of test scores with other variables. The samples included 454 and 179 students for the first and second study, respectively. The data from both studies provided evidence for good psychometric characteristics for the test forms: unidimensionality and local independence, as well as adequate reliability and evidence of validity. The developed test forms are an important contribution in the Portuguese educational context as they allow for the assessment of students’ performance in these skills across multiple time points and can be used both in research and practice.

## Introduction

The product of listening and reading comprehension is an integrated mental representation of the meaning of a text (Oakhill et al., 2019). The processes necessary to extract meaning from written or oral language are generally similar: integration of information, making inferences, association of what one reads/hears with one’s previous knowledge, and construction of the meaning of the material (Perfetti et al., 2005; Cain and Oakhill, 2008).

The assessment of these skills allows us to identify at-risk readers, to support the development of intervention and teaching programs, and to monitor the students’ progress in these areas over time (Santos et al., 2016b). For this purpose, the use of standardized measures with robust psychometric qualities is essential (Salvia et al., 2010).

The overall aim of this paper is to describe the development and validation of two vertically scaled forms of a reading comprehension and a listening comprehension test for Portuguese students in the fifth and sixth grades.

Reading and listening comprehension tests developed to assess specific age or grade level groups are useful tools to compare inter-individual differences (i.e., to compare a student’s performance with a normative group). However, when the goal is to compare the achievement of the same student across different time points (intra-individual differences), the administration of the same test at different educational levels has several disadvantages. The use of the same test across a wide range of academic grades is problematic due to the learning effects and reactivity effects of the measures. Moreover, the results can be influenced by extreme floor or ceiling effects in lower and upper grades, respectively (Santos et al., 2016b). The solution for these problems is to use different and specific test forms for each academic grade with equated scores. Equating is a statistical process that allows the conversion of the scores obtained in different test forms into a single metric, so that these test forms can be used at different points of time and the scores can be directly compared to assess the development of these skills in the same individual over time (Kolen and Brennan, 2014).

Equating models based on item response theory analyses are widely used (Wilson and Moore, 2011). Item response theory analyses, including Rasch model analyses, allow researchers to assess “not only the difficulty level of a specific item, but also permit interval scaling for the assessment of change, assessment of the dimensionality of a set of items, and specification of the range of items (in terms of ‘ability scores’) that characterize a particular measurement device” (Francis et al., 2005, p. 375). Item response theory analyses also allow researchers to perform differential item functioning (DIF) analyses. Differential item functioning ensures equity in testing because identifying items that favor one group over another on a test prevents bias in the comparison of test scores between different groups (Walker and Beretvas, 2001). The development of standardized tests following item response theory, specifically based on Rasch model, has several advantages, such as allowing the selection of the most appropriate items to the level of competence of the group that is intended to evaluate and performing the vertical equating of different versions of the same test (Prieto and Delgado, 2003; Kolen and Brennan, 2014).

Collecting empirical evidence of validity is also crucial for the development of tests. According to the American Educational Research Association et al. (2014), “validity refers to the degree to which evidence and theory support the interpretations of test scores for proposed uses of tests” (p. 11). Evidence based on relationships with other variables is one of the sources of validity evidence and refers to “the degree to which these relationships are consistent with the construct underlying the proposed test score interpretations” (American Educational Research Association et al., 2014, p. 16). It implies the identification of relevant variables for the construct to be measured and the analysis of the relationships between them. Reading comprehension requires the development of basic reading skills, such as oral reading fluency. A fluent reading ability is mandatory so that higher-level processes of reading comprehension can take place. Therefore, medium-to-large correlation coefficients between these skills have been found across a wide range of orthographies with varying depths, in students up to the sixth grade (Yovanoff et al., 2005; Padeliadu and Antoniou, 2014; Fernandes et al., 2017). However, as the automaticity of the basic reading processes increases across schooling years, successful text comprehension becomes more dependent on higher order skills, such as vocabulary, memory, reasoning, and comprehension monitoring (Yovanoff et al., 2005; Sesma et al., 2009; Ouellette and Beers, 2010; Ribeiro et al., 2015a; Nouwens et al., 2016; Fernandes et al., 2017).

With regard to listening comprehension, given that it involves linguistic processes similar to the ones used in reading comprehension, similar results have been observed for the relationship between listening comprehension, vocabulary, and working memory (Ouellette and Beers, 2010; Florit et al., 2014; Tighe et al., 2015; Kim, 2016; Jiang and Farquharson, 2018).

Analogical reasoning also seems to play an important role in solving comprehension tasks, since it enables processes for making inference (Tzuriel and George, 2009). In this sense, previous studies have shown that verbal and non-verbal reasoning had medium-to-large sized correlations with reading and listening comprehension in several orthographies (Ribeiro et al., 2015a; Tighe et al., 2015; Potocki et al., 2017).

Moreover, readers who can successfully comprehend the text employ planning strategies (e.g., evaluate the text’s difficulty before reading) to begin reading metacognitively, and monitoring strategies (e.g., summarize information in the text) to make sense of what they read (Botsas, 2017). However, empirical studies seem to yield mixed results when the use of reading strategies is assessed by self-report measures. For example, in a sample of Croatian students from the fifth to eighth grades, perceived use of reading strategies was significantly associated with reading comprehension only in eighth-grade students (Kolić-Vehovec and Bajšanski, 2006). However, in another study conducted with Chinese students, perceived use of reading strategies was also moderately correlated with reading comprehension among fifth graders (Law, 2009).

Finally, previous studies have also found medium-to-large correlation coefficients between teachers’ ratings of students’ reading skills and students’ performance on standardized tests that assess reading and listening comprehension from kindergarten to the fifth grade (Feinberg and Shapiro, 2003, 2009; Gilmore and Vance, 2007; Viana et al., 2015; Santos et al., 2016a).

## The Present Study

Various measures of reading assessment for elementary school students have been developed in Portugal. One of these measures was the Battery of Reading Assessment (Santos et al., 2015, 2016b), which is composed of vertically scaled forms to assess word reading, listening, and reading comprehension from the first to the fourth grade. The special attention paid to the lower grades of elementary schools for assessing reading and listening comprehension can be explained by the importance and the impact of learning across the primary school years on the subsequent years. However, results from national level reports in Portugal have shown that the number of children who have reading difficulties past lower grades of elementary school is still high (Monteiro et al., 2014). These data raise growing concerns about reading difficulties emerging in the later years of schooling: students who succeed in learning to read in the primary grades, but then fall behind in the upper elementary or middle school grades (Leach et al., 2003; Catts et al., 2005; Lipka et al., 2006). This phenomenon imposes the need for the development of robust measures that not only allow further development of research in this field, but also help to assess and monitor comprehension performance beyond lower elementary school grades. Therefore, the present study intended to expand the Battery of Reading Assessment for fifth and sixth graders in Portugal.

This paper reports the procedure and results of two studies. The purpose of the first study was to develop listening and reading comprehension tests, with two vertically scaled test forms for European Portuguese students in the fifth and sixth grades, using Rasch model analyses. The second study aimed to collect validity evidence for the two vertically scaled forms of each test based on the relationship of test scores to other variables by analyzing the relationships between the developed test forms and measures used as external criteria for oral reading fluency, vocabulary, working memory, comprehension monitoring, verbal and abstract reasoning, teachers’ ratings, and academic achievement. Based on the research literature, it was expected that the scores on the test of reading comprehension will be positively correlated with all the other variables and that the scores on the test of listening comprehension will be correlated with measures of vocabulary, working memory, verbal and abstract reasoning, teachers’ ratings, and academic achievement.

## Study 1

### Materials and Methods

#### Participants

All participants were native speakers of European Portuguese, attending schools located in northern Portugal. The sample included 222 fifth graders (*M*_age_ = 10.95 years, *SD* = 0.58; 52.3% were boys; 77% were attending public schools) and 232 sixth graders (*M*_age_ = 11.98 years, *SD* = 0.42; 52.6% were boys; 89.2% were attending public schools). Students who qualified for educational intervention at the selective and/or additional levels were not included in the sample. With regard to socioeconomic status, 43.7% of the fifth graders and 26.7% of the sixth graders benefited from scholar social support (i.e., reduced-price meals at school, access to a loan service for books, and support for the acquisition of school supplies). Regarding maternal education of the fifth graders, 16.2% of the mothers had completed a university degree, 28.8% had completed high school, and 55% had a lower educational degree. In relation to the sample of sixth graders, 30.2% of their mothers had completed a university degree, 27.6% had completed high school, and 37.9% had a lower educational degree (4.3% missing information).

#### Study Design and Measures

Non-equivalent groups with anchor test design is the most appropriate equating procedure in the construction of measures involving at least two groups that differ in ability level, responding to different test forms (de Ayala, 2009; Kolen and Brennan, 2014). For this purpose, test forms should include a set of common items between adjacent grades, and a set of unique items for each test form, that allows researchers to calibrate each test form separately as well as sequentially using vertical equating (Kolen and Brennan, 2014). This kind of equating is used when groups of subjects differ in ability level and tests differ in level of difficulty (Baker, 1984). This technique is used when the goal is to compare performances in skills that are expected to develop over time, such as listening and reading comprehension (de Ayala, 2009). Specific test forms for each grade were developed to assess reading and listening comprehension at the end of fifth and sixth grades, namely, the Test of Reading Comprehension of Narrative Texts (TRC-n_5/6_) and the Test of Listening Comprehension of Narrative Texts (TLC-n_5/6_). Each test form included a booklet with three original texts (two unique texts for each grade, and one common text between adjacent grades) authored by Portuguese writers of literature for children and a worksheet containing the test items. The length of the texts for TRC-n_5_ and TLC-n_5_ ranged from 551 to 882 words, and for TRC-n_6_ and TLC-n_6_ from 574 to 700 words. Each test form comprised unique and common items between the test forms for the adjacent grades (see Table 1). Regarding TRC-n_5_ and TLC-n_5_, anchor items (and the respective text) were derived from the test forms previously validated for fourth graders (Santos et al., 2015, 2016b). To select the anchor items to be included in the test forms for the fifth grade, the mean difficulty level of the items of the texts that composed the fourth-grade test forms was computed. The text whose items had the highest mean difficulty was selected and the respective items were used as anchor items. Anchor items (and the respective text) of the TRC-n_6_ and the TLC-n_6_ were derived from the TRC-n_5_ and the TLC-n_5_, respectively. Test items were multiple-choice questions with three options. Prior studies have shown that three options are optimal for multiple-choice items, being as psychometrically efficient as four or five options (Delgado and Prieto, 1998; Rodriguez, 2005). Items were developed to assess four levels of comprehension (literal, inferential, critical, and reorganization) described in the taxonomy by Català et al. (2001), that was used in the development of the test forms for primary school students (Santos et al., 2015, 2016b). The items and the options were developed by the researchers and were later revised by text comprehension experts, who have extensive experience in teachers’ training. In the TRC-n, the student silently reads the text passages that are followed by multiple-choice questions and marks the chosen option on the answer sheet (pencil-and-paper format). In the TLC-n, the student listens to the texts divided in short passages and the respective items that are only presented orally though an audiotaped recording and marks the chosen option on the answer sheet. The testing procedure included two example items for all test forms. There was no time limit to complete each test. In the TLC-n_5/6_, the students listened to the texts divided in short passages and the respective items that are only presented orally though an audiotaped recording. The audiotaped recording was stopped after each item so that all students had time to mark their response. The presentation of the next item proceeded only after all students have marked the chosen option on their answer sheet or decided not to respond.

**TABLE 1:** Items in each test form of the TRC-n and TLC-n.

Test form	Initial pool of items							Final pool of items						
		
	LC	IC	CC	R	U	A	T	LC	IC	CC	R	U	A	T
TRC-n_5_	7	21	3	8	32	7	39	6	15	3	8	26	6	32
TRC-n_6_	13	22	3	8	32	14	46	7	14	2	7	22	8	30
TLC-n_5_	12	25	3	8	35	13	48	10	16	2	7	27	8	35
TLC-n_6_	11	19	3	6	27	12	39	7	15	3	4	23	6	29

*LC, number of items of Literal Comprehension; IC, number of items of Inferential Comprehension; CC, number of items of Critical Comprehension; R, number of items of Reorganization; U, number of Unique items; A, number of Anchor items; T, Total of items.*

#### Procedure

Legal authorizations for data collection were obtained from the ethics committee of the University of Minho and the Portuguese Ministry of Education, and from the respective school boards. Informed consent for the test administration was previously collected from parents or legal tutors. The anonymity and confidentiality of the data were guaranteed. Children were informed of the goals and characteristics of the study and were told that they could drop the study at any time. All tests were administered collectively in the classroom by trained psychologists.

### Data Analyses

Ten cases had missing data in the TRL-n_6_, but the number of missing values was only 0.14% of the total data. Five outliers for TRC-n_6_ were found in the exploratory data analyses and were, therefore, removed. Unidimensionality of the test forms was tested using principal component analyses (PCA) of the linearized Rasch residuals. Eigenvalues less than 2.0 and/or explained variance less than 5% for secondary dimensions support this requisite (Linacre, 2018). Correlations between the items’ linearized Rasch residuals were calculated to examine the assumption of local independence of the items. Correlations higher than 0.70 may indicate that response to an item does not exclusively depend on the persons’ ability and is influenced by the performance on another item (Linacre, 2018). The reliability was analyzed by calculating Rasch coefficients for the person- and item-separation reliability (PSR and ISR), as well as Kuder-Richardson formula 20 (KR20). All coefficients values should be higher than 0.70 (Nunnally and Bernstein, 1994). Infit and outfit mean square statistics were analyzed to assess person and item fit to the model. These values should be smaller than 1.5 (Linacre, 2002). Mean ability for the students who selected each answer option was also computed for each item of the test forms. It was expected that the students with highest ability levels would choose the correct options (Linacre, 2018). Differential item functioning analysis was conducted to test the invariance of measurement as a function of sex and socioeconomic status for all items of each test form, using the Rasch-Welch procedure and considering a significance level of 5% (Linacre, 2018). Besides statistical significance, DIF size was also considered for practical significance: it was considered notable if the DIF contrast was ≥| 1.0| logit (Wright and Douglas, 1975, 1976). The displacement of the anchor items was also analyzed in order to evaluate the stability of the common items’ difficulty between adjacent grades. Values of the anchor items’ displacement can assume values as large as 1.0 logits without causing much impact on measurement (Linacre, 2018). The literature also suggests a minimum of 20% of anchor items in tests with 40 or more items for equating purposes (Kolen and Brennan, 2014). Both criteria were taken into account in the decision of deleting anchor items.

The TRC-n and TLC-n forms were linked according to three steps. In a first step, the calibration of the versions for the fifth grade was performed fixing the parameters of the common items with the values obtained in the versions for the fourth grade. In a second step, the items with inappropriate psychometric characteristics were removed. The psychometric characteristics considered were: item misfit, point-measure correlation (correlation between the response to the item and the construct that is being measured by the set of items) lower than 0.15, correct option not chosen by participants with higher levels of the latent trait, presence of DIF as a function of sex and of socioeconomic status, and, in the case of anchor items, displacement higher than 1.0. In a third step, the number of unique items were reduced taken into account the same criteria adopted in the development of the test forms for the primary school students (Santos et al., 2015, 2016b): the spread of difficulty (the items distributed along the continuum of ability of each grade sample were chosen), the redundancy (the number of items of similar difficulty levels was reduced by discarding some redundant items), and the comprehension level (the proportion of items of each comprehension level in the initial pool of items was maintained in the final pool of items, and when two or more items were of similar difficulty levels and they measured the same comprehension level, the item with a higher point-measure correlation was selected). The same steps were followed for the versions of the sixth grade, with the scores of the anchor items obtained in the fifth grade being used in the first step. The test forms were again linked through a final set of calibrations using the unique and anchor items selected in the previous steps. Finally, the reliability coefficients were recalculated. All these analyses were conducted using the Rasch measurement software Winsteps 3.92.1 (Linacre, 2018). Descriptive statistics and one-way analysis of variance to test differences between the grades in the scaled scores obtained on each test form were performed through the statistical program IBM^®^ SPSS Statistics 26. Statistically significant differences were expected in the means between the three grade levels, with successively higher values in upper grade levels.

### Results

#### Dimensionality and Local Independence of the Items

Results of the PCA of the residuals revealed that all the secondary dimensions had eigenvalues close to 2.0 for the initial forms and these secondary dimensions explained less than 5% of the variance (see Table 2). The explained variance by measures was about four times higher than the variance explained by the first secondary dimension. The residuals’ correlations were much lower than 0.70. Therefore, these results support the assumptions of unidimensionality and local independence of the items for implementing Rasch model analyses.

**TABLE 2:** PCA of the residuals and reliability coefficients by test form.

Test form	Highest eigenvalue of the SDi (EV)	RV by measures (%)	Highest correlation of the IR	PSR	KR20	ISR
**Initial pool of items**						
TRC-n_5_	2.17 (4.4%)	21.6	0.25	0.77	0.78	0.97
TRC-n_6_	2.33 (4.0%)	21.0	0.34	0.78	0.80	0.97
TLC-n_5_	2.10 (3.6%)	17.8	0.26	0.73	0.73	0.97
TLC-n_6_	2.29 (4.6%)	21.2	0.23	0.74	0.76	0.97
**Final pool of items**						
TRC-n_5_	1.84 (4.7%)	18.7	0.22	0.75	0.76	0.95
TRC-n_6_	1.92 (4.9%)	24.1	0.18	0.72	0.76	0.97
TLC-n_5_	1.90 (4.5%)	17.1	0.22	0.70	0.72	0.95
TLC-n_6_	2.03 (5.4%)	22.7	0.21	0.70	0.72	0.97

*SDi, Secondary Dimensions; EV, Variance Explained by the secondary dimensions; RV, Raw Variance explained by measures; IR, Items’ Residuals; PSR, Person Separation Reliability; KR20, Kuder-Richardson 20; ISR, Item Separation Reliability.*

#### Item Analyses

Table 3 presents descriptive statistics for the Rasch estimated parameters for each test form.

**TABLE 3:** Descriptive statistics of the estimated parameters by test form.

Test form	Person ability				Item difficulty				Item infit				Item outfit				% person misfit	
					
	*M*	*SD*	Min	Max	*M*	*SD*	Min	Max	*M*	*SD*	Min	Max	*M*	*SD*	Min	Max	Infit > 1.5 (%)	Outfit > 1.5 (%)
**Initial pool of items**																		
TRC-n_5_	1.17	0.78	–0.85	3.19	0.71	0.89	–0.81	3.56	1.00	0.07	0.89	1.14	1.00	0.12	0.83	1.47	0.5	4.5
TRC-n_6_	2.03	0.85	–0.39	5.13	0.84	0.99	–1.92	3.74	1.01	0.14	0.71	1.65	0.97	0.26	0.49	2.14	0	4.3
TLC-n_5_	1.86	0.66	0.17	3.56	1.10	0.89	–0.74	3.91	1.02	0.14	0.66	1.51	1.03	0.17	0.64	1.60	0	2.3
TLC-n_6_	2.46	0.82	0.02	4.64	1.31	0.99	–0.64	3.27	1.02	0.10	0.88	1.27	0.99	0.15	0.66	1.27	0.9	5.2
**Final pool of items**																		
TRC-n_5_	1.17	0.83	–1.20	3.55	0.64	0.69	–0.61	1.85	1.00	0.08	0.89	1.16	0.99	0.11	0.83	1.27	0	0.5
TRC-n_6_	2.08	0.98	–0.53	4.67	0.70	1.09	–1.98	3.96	1.01	0.12	0.71	1.37	0.97	0.24	0.55	1.49	0	7.0
TLC-n_5_	2.03	0.78	0.26	4.05	0.99	0.75	–0.64	3.04	1.01	0.10	0.68	1.40	1.01	0.13	0.66	1.43	0	2.3
TLC-n_6_	2.50	0.90	–0.49	5.10	1.35	0.95	–0.30	3.33	1.00	0.09	0.89	1.28	0.97	0.15	0.68	1.24	0.7	6.5

*M, Mean; SD, Standard Deviation; Min, Minimum; Max, Maximum.*

##### Tests of Reading Comprehension

In the TRC-n_5_ none of the items exceeded the reference value of 1.5 for infit and outfit statistics (see Table 3) and the highest mean ability value was obtained by students who chose the correct answer option for all 39 items. However, one item exhibited a difficulty value higher than the maximum value for person ability, meaning that it was too difficult for fifth graders. The same item presented a point-measure correlation lower than 0.15. Moreover, four items were flagged with DIF as a function of sex and two items were flagged with DIF as a function of socioeconomic status. Two of these six items were anchor items. Consequently, only one out of these two (the one with the highest DIF contrast) was eliminated in order to maintain the percentage of anchor items close to the minimum value of 20%. The item that was maintained in the measure obtained a DIF contrast of 0.65 and, therefore, its impact was considered not notable. In addition to the six items with inappropriate psychometric characteristics mentioned above, one more item was deleted according to the criteria for selection of unique items. Therefore, seven items were removed from the initial version of TRC-n_5_. Thus, the final version of TRC-n_5_ was composed of 32 items with six anchor items (18.8% of the total number of items).

**FIGURE 1:**
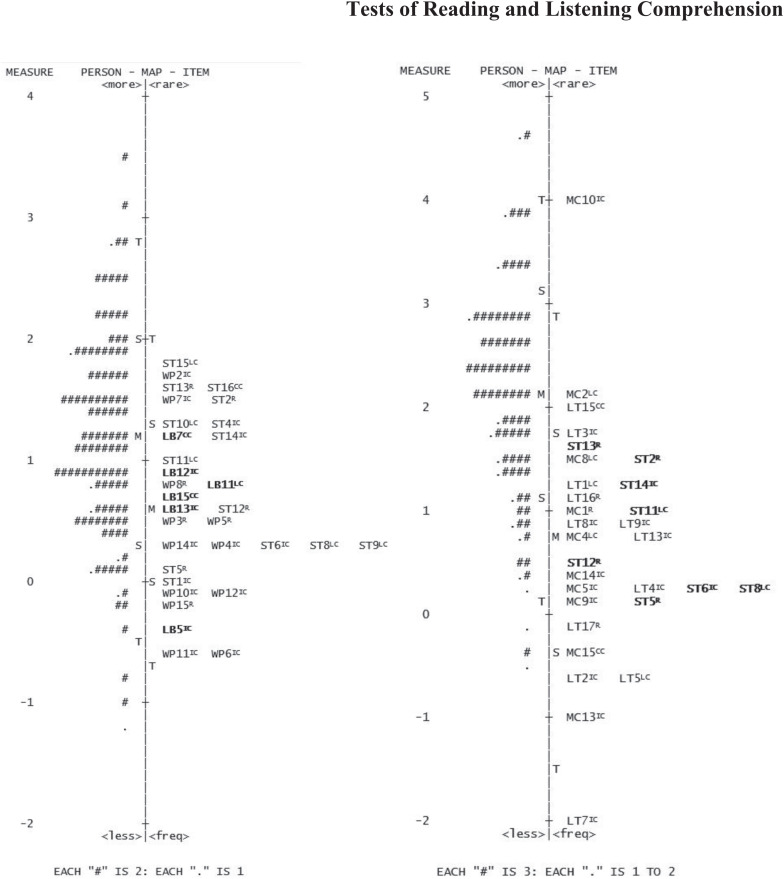
Person-item variable map for TRC-n_5_ (left) and TRC-n_6_ (right). Items are identified by the text to which they are related (WP, The history of white pencil; ST, A very special trip; LB, The lost bread; MC, A mysterious chest; LT, Loose thoughts), followed by the item’s number. The comprehension level assessed by each item is presented in superscript (LC, Literal Comprehension; IC, Inferential Comprehension; R, Reorganization; CC, Critical Comprehension). Anchor items appear in bold.

In the TRC-n_6_ initial pool of 46 items, four items presented difficulty levels lower than the minimum person ability value, meaning that they were too easy for fifth graders. Further, one item had infit and outfit values higher than 1.5 and a negative point-measure correlation. In the same item, students who chose the correct answer option were not the ones with the highest average ability levels. A second item had a point-measure correlation lower than 0.15. This one and more two items were also flagged with DIF as a function of sex. Additionally, four items were flagged with DIF as a function of socioeconomic status. Therefore, eight items were removed from TRC-n_6_. According to the criteria for selection of unique items, eight other items were also removed. Therefore, the final version of TRC-n-6 was composed of 30 items with eight anchor items (26.7% of the total number of items). Figure 1 presents the item and person parameter locations in the vertical scale resulting from the final recalibration of the TRC-n_5_ (left) and the TRC-n_6_ (right). Mean values of the person ability standardized scores for the TRC-n were 111 (*SD* = 10) for the TRC-n_5_, and 120 (*SD* = 10) for the TRC-n_6_. In the validation study of the version for the fourth grade (TRC-n_4;_ Santos et al., 2016b), the mean was 108 (*SD* = 10). With the progress in grade levels (lower to higher), person ability values were significantly greater, *F*(2, 670) = 90.874, *p* < 0.001. *Post-hoc* tests revealed significant differences (*p* < 0.001) between the scaled scores obtained on the three TRC-n test forms.

##### Tests of Listening Comprehension

Of the TLC-n_5_ initial pool of 48 items, 6 items exhibited difficulty values lower than the minimum value for person ability (see Table 3), meaning that they were very easy for fifth graders. Additionally, one item exhibited difficulty value higher than the maximum value for person ability, indicating that it was very difficult for fifth graders. Regarding the infit and outfit statistics, two items exceeded the reference value of 1.5. Three items had point-measure correlations lower than 0.15. In one of these three items, the highest mean ability value was not obtained by students who chose the correct answer option, suggesting that the students with greater reading comprehension abilities chose an incorrect alternative. Additionally, four items were removed because they were flagged as having DIF regarding sex or socioeconomic status. According to the criteria for the selection of unique items, four additional items were also removed. Therefore, a total of 13 items were removed. Thus, the final version of the TLC-n_5_ was composed of 35 items with eight anchor items (22.9% of the total number of items).

**FIGURE 2:**
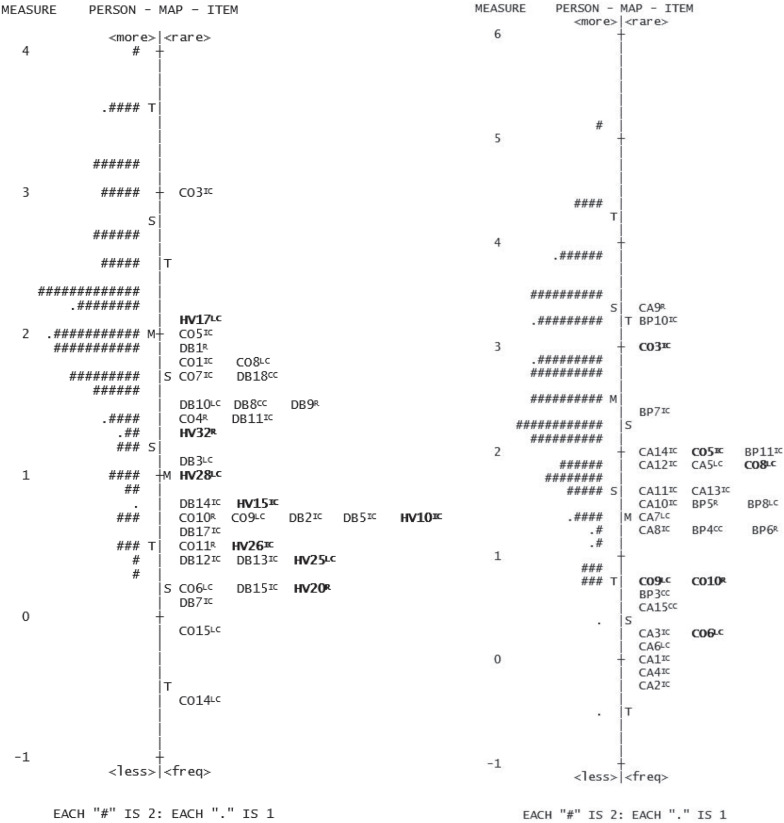
Person-item variable map for TLC-n_5_ (left) and TLC-n_6_ (right). Items are identified by the text to which they are related (DB, The Dentuça Bigodaça; CO, A composition; HV, Holidays in the Village; CA, The camping; BP, The bird and the pine), followed by the item’s number. The comprehension level assessed by each item is presented in superscript (LC, Literal Comprehension; IC, Inferential Comprehension; R, Reorganization; CC, Critical Comprehension). Anchor items appear in bold.

In the TLC-n_6_ initial pool of 39 items, the minimum person ability value exceeded the minimum value of items difficulty for four items, meaning that these items were easy for sixth graders. Regarding the fit statistics for the items, none of the items exceed the reference value of 1.5. Only one item had point-measure correlation lower than 0.15. Two items were flagged as having DIF both as a function of sex and socioeconomic status and, therefore, were eliminated. Additionally, four items were flagged as having DIF only as a function of sex and six as having DIF only as a function of socioeconomic status. Only seven out of these 10 items were removed in order to maintain acceptable reliability coefficients (PSR, KR20 and ISR). The three with the lowest DIF contrast, ranging between 0.61 and 0.78, were maintained in the test form. As a summary, 10 items were removed and the final version of the TLC-n_6_ was composed of 29 items, among which six were anchor items (20.69% of the total number of items). Figure 2 presents the item and person parameter locations in the vertical scale resulting from the final recalibration of the TLC-n_5_ (left) and the TLC-n_6_ (right). Mean values of the person ability standardized scores for the TLC-n were 124 (*SD* = 10) for the TLC-n_5_, and 128 (*SD* = 10) for the TLC-n_6_. In the validation study of the version for the fourth grade (TLC-n_4;_ Santos et al., 2015) the mean was 122 (*SD* = 10). With the progress in grade levels (lower to higher), person ability values were significantly greater, *F*(2, 711) = 24.721, *p* < 0.001. *Post-hoc* tests revealed significant differences (*p* < 0.05) between the scaled scores obtained on the three TLC-n test forms.

#### Reliability

The PSR and KR20 values were moderate, and the ISR coefficients were very high for the initial and final versions of all test forms. The elimination of items from the initial test forms to the final test forms did not cause a sharp decrease in reliability (see Table 2).

## Study 2

### Materials and Methods

#### Participants

A group of 179 students participated in the study of validity evidence of the TRC-n and the TLC-n forms: 94 were fifth graders (*M*_age_ = 10.96 years, *SD* = 0.55; 52.1% were girls) and 85 were sixth graders (*M*_age_ = 12.01 years, *SD* = 0.50; 52.9% were boys). All students attended public schools located in northern Portugal and were native speakers of European Portuguese. Students with special educational needs who were identified for selective and/or additional intervention were not included in the sample.

#### Measures

##### TRC-n_5/6_ and TLC-n_5/6_

In this study, the final versions of these scales as developed in Study 1, were used.

##### Test of Reading Fluency

This test assesses oral reading fluency in students from the first to sixth grade, with an unpublished text composed of 1,160 words. Students were asked to read the text aloud within 3 min. Word omissions, substitutions, and mispronunciations were scored as errors. Self-corrections within 3 s after the error, repeated words, mispronunciations due to dialect or regional differences, hesitations, or words read slowly but correctly were not scored as errors. The number of words read correctly per minute was calculated using the mean of the 3 min.

##### Vocabulary Subtest of the Wechsler Intelligence Scale for Children-III (WISC-III; Wechsler, 2003)

It has 30 items composed of words presented orally which the students were asked to define orally, as completely as possible. Each item can be scored with zero, one, or two points, depending on the quality of the response, and the raw scores are converted to standardized scores. The administration is interrupted after four consecutive failures. High reliability and good indicators of validity were found in the Portuguese version of the WISC-III (Simões and Albuquerque, 2002).

##### Digit Span Subtest of the WISC-III (Wechsler, 2003)

This subtest includes two tests of digit span (forward and backward). Each series consists of two rehearsals and the test is interrupted after both rehearsals of the same series fail. Items are scored with zero or one point, and the raw scores were converted to standardized scores. Reliability coefficients for the Portuguese Digit Span subtest ranged between 0.71 and 0.90 (Simões and Albuquerque, 2002).

##### Reading Strategy Use (Ribeiro et al., 2015b)

This scale is composed of 22 items that assess cognitive and metacognitive strategy use (10 and 12 items, respectively). Each item consists of a proposition that represents a reading strategy, and the student’s task is to mark the frequency of its use on a 7-point Likert scale ranging from 1 (*never*) to 7 (*always*). The adaptation and validation studies of this scale for the Portuguese population supported the one-dimensional structure assumption. Cronbach’s alpha coefficient was 0.85 for the 22 items (Ribeiro et al., 2015b).

##### Verbal Reasoning Subtest (Almeida and Lemos, 2006)

This subtest assesses the ability to infer and apply relationships using tasks of verbal content. It consists of 20 multiple-choice items with four options (one correct), involving analogies between words. The test administration has a time limit of 4 min. Items are scored with zero or one point. Reliability coefficient for this test was 0.72 (Almeida and Lemos, 2006).

##### Abstract Reasoning Subtest (Almeida and Lemos, 2006)

It is composed of 20 items representing analogies with geometrical figures that have to be answered within a 5-min time limit. Items are multiple-choice questions with four options (one correct). Items are scored with zero or one point. Reliability coefficient for this test was 0.71, and statistically significant correlation coefficients were obtained with school achievement in subjects, such as “Portuguese” and “mathematics” (Almeida and Lemos, 2006).

##### Teacher Ratings of Students’ Reading Skills

Teachers were asked to rate students’ performance in oral reading fluency, listening, and reading comprehension in the classroom, using a scale ranging from 1 (*very poor*) to 5 (*excellent*).

##### Academic Results

Two scores of academic achievement were collected: the classification in the subject “Portuguese” and the grade point average obtained at the end of the academic year.

#### Procedure

Similar procedures to the ones used in Study 1 were employed. The TRC-n_5/6_, TLC-n_5/6_, both subtests of reasoning and the strategy use scale were administered collectively, while the remaining tests were administered individually.

#### Data Analyses

Descriptive statistics and Pearson and Spearman correlation coefficients between all variables were calculated using IBM^®^ SPSS Statistics 26. The size of the correlations was evaluated according to the following criteria: 0.10, 0.30, and 0.50 indicates a small, a medium, and a large effect, respectively (Cohen, 1992).

## Results

Table 4 provide the descriptive statistics and the correlations between the scores on the TRC-n_5/6_ and TLC-n_5/6_ and the other measures used as external criteria for the students’ abilities.

**TABLE 4:** Descriptive statistics and correlations in the fifth and the sixth grades.

Tests	*M_grade5_*	*SD_grade5_*	*M_grade6_*	*SD_grade6_*	1	2	3	4	5	6	7	8	9	10	11	12	13
1. TRC-n	111.30	10.99	116.58	11.87	–	0.57***	0.54***	0.77***	0.26*	0.16	0.21	0.20	0.62***	0.55***	0.65****	0.55***	0.62***
2. TLC-n	124.14	10.63	124.67	9.63	0.55***	–	0.35***	0.53***	0.18	0.10	0.26*	0.22*	0.49***	0.39***	0.45***	0.46***	0.53***
3. TRF	126.84	23.38	137.40	25.60	0.37***	0.18	–	0.48***	0.24*	0.12	0.14	0.13	0.52***	0.48***	0.45***	0.39***	0.51***
4. V	9.88	3.13	9.70	2.98	0.53***	0.49***	0.34***	–	0.39***	0.13	0.16	0.17	0.61***	0.55***	0.64***	0.61***	0.65***
5. DS	9.53	3.03	8.80	2.39	0.31**	0.31**	0.22*	0.32**	–	–0.04	0.18	0.13	0.30**	0.14	0.16	0.16	0.19
6. RSU	102.28	21.60	99.18	18.18	0.42***	0.15	0.34***	0.14	0.05	–	–0.13	0.00	0.07	0.12	0.10	0.10	0.10
7. AR	12.70	3.09	13.51	2.78	0.49***	0.45***	0.14	0.38***	0.34***	0.11	–	0.48***	0.09	0.05	0.10	0.06	0.09
8. VR	12.59	3.36	13.80	3.12	0.54***	0.36***	0.37***	0.32**	0.23*	0.22*	0.44***	–	0.22*	0.12	0.17	0.11	0.17
9. GPA	3.66	0.70	3.82	0.73	0.59***	0.58***	0.35***	0.45***	0.32**	0.27**	0.34***	0.45***	–	0.45***	0.48***	0.61***	0.75***
10. ORF	3.44	0.90	3.56	0.92	0.57***	0.52***	0.62***	0.50***	0.21*	0.28**	0.36***	0.42***	0.57***	–	0.79***	0.77***	0.65***
11. LC	3.60	0.79	3.41	0.88	0.58***	0.45***	0.59***	0.55***	0.25*	0.17	0.41***	0.51***	0.53***	0.76***	–	0.82***	0.55***
12. RC	3.30	0.87	3.24	0.92	0.64***	0.58***	0.54***	0.51***	0.23*	0.22*	0.37***	0.45***	0.51***	0.72***	0.78***	–	0.53***
13. PC	3.42	0.77	3.53	0.67	0.53***	0.55***	0.43***	0.44***	0.22*	0.17	0.35***	0.47***	0.74***	0.61***	0.57***	0.71***	–

TRC-n, Test of Reading Comprehension of Narrative Texts, TLC-n, Test of Listening Comprehension of Narrative Texts; TRF, Test of Reading Fluency; V, Vocabulary; DS, Digit Span; RSU, Reading Strategy Use; AR, Abstract Reasoning; VR, Verbal Reasoning; GPA, Grade Point Average; ORF, Oral Reading Fluency assessed by teachers; LC, Listening Comprehension assessed by teachers; RC, Reading Comprehension assessed by teachers; PC, Classification in the subject of “Portuguese” at the final of academic year. Correlation coefficients below the diagonal are for students in the fifth grade and above the diagonal are for students in the sixth grade. The first nine correlation coefficients for each grade are Pearson correlations; the remaining four correlation coefficients are Spearman correlations. ***p < 0.001, **p < 0.01, *p < 0.05.

High correlations were found between the TRC-n and TLC-n test forms in both grades. The correlations between the TRC-n forms and oral reading fluency assessed with the TRF, were moderate in the fifth grade and high in the sixth grade. The scores on the TRC-n were also moderately correlated with the use of reading strategies in the fifth grade, but not in the sixth. All of the TRC-n and TLC-n test forms were highly correlated with measures of vocabulary and with academic results of the students. The correlations of the TRC-n and the TLC-n with measures of working memory and abstract reasoning were moderate in the fifth grade and low in the sixth grade. Correlation coefficients with verbal reasoning were medium-to-large in the fifth grade, but small in the sixth grade. Moderate-to-high correlations with teacher ratings were found for all test forms in both grades.

## Discussion

The aim of the first study was to develop vertically scaled test forms for listening and reading comprehension for Portuguese students in the fifth and sixth grades, through the application of Rasch model analyses. In the second study, evidence for the validity of these forms was collected, based on relationships of the test scores with other variables.

Regarding the first study, the selection of items for each form of the TRC-n and TLC-n took into account the items with misfit, point-measure correlations lower than 0.15, unexpected responses (i.e., the highest mean ability value was obtained by students who chose an incorrect answer option), and the presence of DIF. Items flagged with any of these problems were removed from the respective test forms. Anchor items with high displacement values were also eliminated from each test form. Unique items for each test form were selected by considering the same criteria used in the development of the TRC-n and TLC-n for primary school, that is, first to fourth grades, in previous studies (Santos et al., 2015, 2016b). The TRC-n_5_ and the TRC-n_6_ were composed of 32 and 30 items, with six and eight anchor items, respectively. Regarding the TLC-n, the test form for the fifth grade included 35 items, 8 of which were anchor items, and the test form for sixth grade included 29 items, with 6 anchor items. Anchor items represented about 20% of the final pool of items in all test forms, as recommended by Kolen and Brennan (2014). All items from the TRC-n and TLC-n final forms revealed appropriate psychometric characteristics. In order to maintain this percentage of anchor items in the final versions, one item with socioeconomic status-related DIF was maintained in the TRC-n_5_. Additionally, to maintain acceptable reliability, another three items with DIF (one with gender-related DIF and two with socioeconomic status-related DIF) were retained in the TLC-n_6_. This number of items with DIF represents a low percentage of the test forms (3.1 and 6.9% respectively), and most likely has a low impact on the validity of test forms’ scores, given that their size was not considered notable, according to Wright and Douglas (1975, 1976) criteria and it is common to have about 15% of items with DIF in achievement tests (Narayanan and Swaminathan, 1994; Buzick and Stone, 2011). *Post-hoc* test results showed that the TRC-n and the TLC-n were able to capture the reading and listening comprehension improvements of students across subsequent grades. Additionally, evidence of adequate reliability was obtained for these final test forms. In summary, the results from the first study provided evidence for good psychometric properties for all forms of the TRC-n and the TLC-n.

Regarding the second study, the TRC-n forms were highly correlated with the TLC-n forms. Correlations of moderate magnitudes between listening and reading comprehension in the same grade levels have been found in other studies (Diakidoy et al., 2005; Ouellette and Beers, 2010; Tobia and Bonifacci, 2015). The close relationship between reading and listening comprehension is congruent with the idea that cognitive processes involved in both skills are the same (Perfetti et al., 2005; Cain and Oakhill, 2008). Additionally, most correlations between the developed test forms and external criteria were positive and statistically significant. Moderate to high correlations between the TRC-n and the oral reading fluency measure are consistent with findings from other studies with samples of speakers of a wide range of languages and orthographies, enrolled in the fifth and sixth grades (Yovanoff et al., 2005; Padeliadu and Antoniou, 2014; Fernandes et al., 2017). The TRC-n was moderately correlated with reading strategy use in the fifth grade. This result is particularly similar to the one obtained by Law (2009). In contrast, in the sixth grade, this relationship was not statistically significant, similar to the results reported in the study by Kolić-Vehovec and Bajšanski (2006). These mixed findings can be explained by a decrease of the influence of the use of reading strategies on reading comprehension with progress in schooling from lower to higher grades, when other variables, such as vocabulary gain more influence on reading comprehension (Ouellette and Beers, 2010). Congruent with this idea, the results of this study suggest large correlations between vocabulary and the TRC-n and TLC-n forms. Similar results were observed in previous research conducted with fifth and sixth graders (Yovanoff et al., 2005; Ouellette and Beers, 2010; Nouwens et al., 2016; Fernandes et al., 2017). This finding suggests that vocabulary has a strong influence on both reading and listening comprehension in this phase of development.

Most correlations between the TRC-n and TLC-n forms, working memory, and reasoning skills were statistically significant, similar to what has been reported in other studies with students of different ages and countries (Sesma et al., 2009; Ribeiro et al., 2015a; Tighe et al., 2015; Potocki et al., 2017; Jiang and Farquharson, 2018). However, the effect size of these relationships was lower in the sixth grade compared to the fifth grade. These results might suggest that cognitive variables, such as working memory and reasoning, assume less weight in comprehension as students reach upper elementary and middle school grades.

Finally, medium-to-large correlation coefficients were found between the TRC-n and TLC-n forms and the teachers’ ratings regarding students’ performance in oral reading fluency, listening, and reading comprehension, and high correlations were found with academic achievement indicators. Prior studies reported correlations of similar magnitude between these variables (Feinberg and Shapiro, 2003, 2009; Gilmore and Vance, 2007; Viana et al., 2015; Santos et al., 2016a). These results suggest that the scores in the developed test forms are a fair representation of actual school achievement of the students. Overall, the findings of the second study provide evidence of validity for the developed test forms.

The development of the TRC-n_5/6_ and the TLC-n_5/6_ forms is an important contribution for the assessment of reading and listening comprehension in the Portuguese educational context; these two vertically scaled test forms, used in combination with the test forms developed for primary school (Santos et al., 2015, 2016b), allow for the assessment and monitoring of students’ performance in these skills across multiple time points from the first to the sixth grade, allowing the direct comparison of the scores, and avoiding learning effects. Students that obtained low scores in these tests should be referred to a more comprehensive assessment, including the assessment of cognitive abilities, and to an appropriate intervention. Students may present difficulties in listening comprehension or in reading comprehension, or even in both skills (Nation, 2005). Activities focused on the explicit teaching of vocabulary, the activation of previous knowledge, the teaching of comprehension strategies, questioning, comprehension monitoring, making inferences and retelling, are usually effective in the promotion of comprehension skills in both modalities (oral or written) (Snowling and Hulme, 2012; Hogan et al., 2014). Moreover, given that different types of comprehension are assessed, the results in these tests can also inform instructional decision-making, by indicating which types—literal, inferential, reorganization, and/or critical comprehension—need further attention. For example, students might be able to respond correctly to questions that require only the comprehension of the information explicitly stated in the text but not be able to make inferences when asked to. In this case, teachers can design lessons to foster inferential comprehension, including strategies, such as the expansion, activation and mobilization of relevant previous knowledge or strategies targeting the integration of in-text and out-of-text information (Barth and Elleman, 2017).

These tests are also adequate tools for large-scale testing in schools. Large-scale testing can positively affect different stakeholders. Abu-Alhija (2007) pointed their benefits for students, teachers, parents, administrators and policymakers. Regarding the students, large-scale testing results can encourage students to work more efficiently by describing their knowledge at the time of the assessment and by flagging what needs to be further studied and learned. Teachers can also benefit of the use of this type of tests because large-scale assessments can help to recognize strengths/weakness in the curriculum, to detect deficit areas in the students’ knowledge and to redirect instruction. Moreover, this information can motivate teachers to invest in their professional development and to enhance instruction. One of the possible positive effects of large-scale testing in parents is to stimulate their involvement in school activities. The use of these tests can also help administrators, for example, in the evaluation of the qualities of their programs. Additionally, large-scale testing can help policymakers’ in the evaluation of the effectiveness of the educational polices and in the promotion of a better allocation of resources (Abu-Alhija, 2007).

This study also had certain limitations, such as the use of a convenience sampling technique and the centralization of the data collection in northern Portugal. Thus, the results of the study should be generalized with caution. Another limitation was the maintenance of some items that showed DIF. Although the size of the differences was not very large, these items can introduce some bias in testing, especially when comparing the results of students from different socioeconomic levels.

The collection of evidence based on consequences of testing can be an important aim for future studies. The administration of tests in educational contexts is based on the idea that the interpretation of the scores should be used for realizing some benefits, such as improvement of learning and motivation for students through the selection of efficacious instructional strategies. Interviews and focus groups with teachers and students as well as classroom observations can be used to obtain data about this type of validity. Further research may analyze the accuracy classification indices of these tests in identifying students with reading difficulties in the fifth and sixth grades. Taking into account that prior literature has shown that different results can be obtained for comprehension tests with texts of distinct typologies (RAND Reading Group, 2002), future research may also focus on development of the TRC-n and the TLC-n for the same grade levels using expository texts. Consequently, the separate or combined use of reading and listening comprehension tests with narrative and expository texts may contribute to the identification of specific comprehension difficulties and then guide intervention programs more centered on training strategies for extracting meaning from texts of one or both typologies.

## Author’s Note

IC was in the Research Centre on Child Studies at the time of the study and is now in the Psychology Research Centre of the University of Minho.

## Data Availability Statement

The raw data supporting the conclusions of this article will be made available by the authors, without undue reservation, to any qualified researcher.

## Ethics Statement

This study was reviewed and approved by the Ethics Committee of the University of Minho, the Portuguese Ministry of Education and the school boards involved. Written informed consent was collected from children’s parents or other legal tutors.

## Author Contributions

BR made substantial contributions to the conception and design of the study, data collection, statistical data analysis and interpretation, and discussion of the results. IC contributed to the design of the study, statistical data analysis and to the interpretation, and discussion of the results. FV and IR made substantial contributions to the conception and design of the study, elaboration of the tests’ content, and interpretation and discussion of the results. All authors were involved in drafting the manuscript and revising it critically for important intellectual content.

## Conflict of Interest

The authors declare that the research was conducted in the absence of any commercial or financial relationships that could be construed as a potential conflict of interest.
